# Radioelectric Asymmetric Conveyer (REAC) Neuropostural Optimization (NPO) for Pain in Lipedema: A Sham-Controlled Study

**DOI:** 10.7759/cureus.92331

**Published:** 2025-09-14

**Authors:** Vania Fontani, Arianna Rinaldi, Alessandro Castagna, Salvatore Rinaldi

**Affiliations:** 1 Department of Research, Rinaldi Fontani Foundation, Florence, ITA; 2 Department of Regenerative Medicine, Rinaldi Fontani Institute, Florence, ITA; 3 Department of Adaptive Neuro Psycho Physio Pathology and Neuro Psycho Physical Optimization, Rinaldi Fontani Institute, Florence, ITA

**Keywords:** epigenetic stress, functional dysmetria, lipedema, neuromodulation, neuropostural optimization (npo), noninvasive treatment, pain reduction, radioelectric asymmetric conveyer (reac), sham-controlled study, visual analog scale (vas)

## Abstract

Background: Lipedema is a chronic disorder characterized by symmetrical and disproportionate fat accumulation, pain, and easy bruising, often resistant to conventional treatments. Functional dysmetria (FD), a maladaptive neuromotor response linked to epigenetic stress, has been proposed as a relevant contributor to pain in lipedema.

Objective: This retrospective observational study aimed to evaluate whether correcting FD through the radioelectric asymmetric conveyer (REAC) technology Neuropostural Optimization (NPO) protocol can reduce pain in patients with lipedema, using a sham-controlled design.

Methods: In this retrospective observational study, 83 consecutive women with stage 2-5 lipedema underwent both sham and real NPO procedures during a single session. Pain intensity was measured using the visual analog scale (VAS) immediately after each procedure. Statistical analyses were performed using paired t-tests with effect sizes (Cohen’s dz) and 95% confidence intervals.

Results: Baseline pain was uniformly high (mean 7.41 ± 0.53), with some pre-sham values reaching 10/10. Pain reduction was observed exclusively after real NPO and not after sham. Sham NPO induced no significant changes, whereas real NPO yielded a mean pain reduction exceeding 3.5 VAS points across all stages (mean change -3.65 ± 0.62; 95% CI: -3.79 to -3.51; Cohen’s dz = 5.88, 95% CI: 4.85-6.92; p < 0.0001).

Conclusions: These findings suggest that correction of FD by REAC NPO provides rapid and clinically meaningful analgesic effects in lipedema patients, addressing an upstream neuropsychomotor dysfunction that may contribute to disease progression. However, given the retrospective single-center design and absence of long-term follow-up, these conclusions are limited to the short-term effects observed. Further multicenter and longitudinal studies are warranted to confirm these results and evaluate long-term outcomes.

## Introduction

Functional dysmetria (FD) is a neuropsychophysical condition present from birth, characterized by asymmetric and maladaptive postural responses to environmental stimuli [1]. In the context of lipedema, these maladaptive responses may exacerbate microvascular congestion, lymphatic overload, and tissue inflammation, thereby aggravating pain and disease progression [2,3]. This retrospective observational study investigates whether correcting FD through the radioelectric asymmetric conveyer (REAC) technology Neuropostural Optimization (NPO) protocol [4] can yield measurable benefits in pain reduction for women with lipedema.

Lipedema [5] is a chronic, progressive disorder of subcutaneous adipose tissue that predominantly affects women [6]. It is characterized by symmetrical and disproportionate fat accumulation in the lower and, less frequently, upper limbs, accompanied by pain, tenderness, and a tendency to bruise easily [7]. Although first described decades ago, lipedema remains underdiagnosed and frequently misinterpreted as obesity or lymphedema [8]. Its etiology is multifactorial, involving genetic predisposition, hormonal influences, particularly estrogen-mediated effects, and microvascular and lymphatic dysfunction [9]. The chronic pain associated with lipedema is one of the most debilitating symptoms, often severely impacting patients’ quality of life, mobility, and psychological well-being [10].

Traditional management strategies for lipedema focus primarily on symptom control and slowing disease progression. These include conservative measures such as manual lymphatic drainage, compression therapy, exercise, and dietary modifications [9,11]. While such approaches may provide transient relief from edema or improve limb contour, their effectiveness in addressing chronic pain is limited [12]. Many patients, including those in advanced stages of the disease, report minimal or no improvement in pain despite sustained adherence to these therapies. Surgical options, such as liposuction, can reduce fat deposits but are invasive, associated with significant recovery times, and not always successful in relieving pain [13,14].

Given these limitations, there is a pressing need for novel, noninvasive, and safe interventions that target the underlying pathophysiological mechanisms contributing to pain in lipedema. One emerging therapeutic approach is neuromodulation, which seeks to alter dysfunctional neural activity within the central and peripheral nervous systems to restore normal sensory processing and motor control. The REAC technology represents a unique form of neurobiological modulation, delivering asymmetrically conveyed radioelectric fields designed to interact with the body's endogenous bioelectric activity.

Within the REAC platform, the NPO protocol has been developed to address maladaptive postural and motor patterns [1,4,15]. These patterns can be both a cause and a consequence of chronic pain syndromes, as they perpetuate dysfunctional sensory-motor integration and central sensitization. Previous studies have demonstrated that NPO can induce measurable improvements in postural control, reduce functional asymmetries, and modulate symptoms in various chronic conditions [16-18].

In the context of lipedema, pain may be amplified by altered central processing of nociceptive input [19] and by maladaptive motor patterns developed over years of compensatory movement strategies [12]. Therefore, targeting these central mechanisms through NPO could represent an upstream intervention [4], potentially reducing pain irrespective of the local adipose tissue pathology.

The present retrospective observational study was designed to evaluate the analgesic effects of a single NPO session in women with stage 2-5 lipedema who had previously undergone multiple unsuccessful physical and dietary interventions. To control for placebo effects, we incorporated a sham NPO pre-treatment within the same patient group, allowing for direct comparison of immediate pain responses to sham versus active intervention. This approach aimed to provide robust preliminary evidence of the specific effects of NPO on pain perception in this patient population.

## Materials and methods

This was a retrospective observational study of consecutive lipedema patients treated at the Istituto Rinaldi Fontani (Florence, Italy) between January 2023 and June 2025. The sample comprised all consecutive eligible cases during the study period, providing a representative snapshot of the clinical population.

Inclusion criteria

A total of 83 women, aged 26 to 72 years, with a clinically confirmed diagnosis of lipedema at stages 2 to 5, were included. The exclusive inclusion of women reflects the epidemiology of lipedema, which predominantly affects females, with only anecdotal cases reported in men, usually in association with severe endocrine disorders [20]. Thus, the sample represents the clinically relevant population, minimizing potential sex-related confounders. Diagnosis was based on established clinical criteria, including symmetrical and disproportionate fat distribution, chronic pain or tenderness upon palpation, easy bruising, and the exclusion of other conditions such as lymphedema or generalized obesity. Disease stage was determined by clinical evaluation, and baseline pain levels were generally comparable across the cohort. Only a small proportion of patients sought consultation specifically for lipedema; most presented with broader symptoms such as generalized pain, lower limb discomfort, fatigue, or functional impairments. Although comorbidities were not systematically recorded, clinical assessment confirmed the absence of major concurrent conditions that could have confounded pain evaluation. All participants had previously undergone conservative approaches (manual lymphatic drainage, compression garments, physiotherapy, and dietary interventions), with limited or no sustained pain relief.

Study design considerations

Although the retrospective design may be considered a limitation compared to prospective trials, several methodological features strengthen the robustness of the findings. First, the inclusion of all consecutive eligible patients during the study period minimized selection bias and ensured that the sample reflected real-world clinical practice. Second, the use of an internal sham procedure within the same session provided a rigorous control condition, allowing each patient to serve as their own comparator and thereby reducing inter-individual variability. Third, as both real and sham procedures are routinely performed in clinical practice to assess the role of functional dysmetria, the retrospective analysis captured data generated in a standardized and reproducible context. The fixed sequence of sham followed by real NPO was necessary because correction of FD is irreversible within the same session, preventing a crossover design in this setting. Together, these elements enhance the validity of the results and mitigate the potential weaknesses typically associated with retrospective observational studies.

Ethics statement

This retrospective observational study was conducted in accordance with the ethical principles of the Declaration of Helsinki. All procedures consisted of treatments routinely applied in clinical practice, with no experimental interventions or invasive methods. At our institution, the sham NPO procedure was already part of routine diagnostic evaluation in patients with pain-related conditions before the conception of this study and was therefore not introduced for research purposes. The study involved only standard care supported by European Conformity (CE)-certified medical devices (REAC BENE 110). The protocol was reviewed and approved by the Internal Review Board (IRB) of the Rinaldi Fontani Institute (Protocol Number: IRB-RFI-2025-07-1-1). Written informed consent was obtained from all patients.

Intervention protocol

The NPO protocol consists of a single radioelectric asymmetrically conveyed signal lasting a few milliseconds, delivered through a CE-certified REAC medical device using fixed pre-set parameters that cannot be modified by the operator, thereby ensuring standardization and reproducibility across sessions and patients [4]. The procedure is completely imperceptible to the patient, with no reported sensations during administration. Each participant underwent two consecutive sessions, spaced approximately five minutes apart: a sham NPO session followed by the real NPO intervention.

The sham session was identical in all procedural aspects, including Asymmetric Conveyor Probe (ACP) placement, session duration, and operator interaction, but without the delivery of the active REAC signal. Both the patient and the outcome assessor were blinded to the allocation of sham versus real intervention. This within-subject approach allowed each participant to serve as her own control, reducing variability and minimizing potential confounding factors. The real NPO treatment was administered using the REAC BENE 110 device (ASMED, Scandicci, Italy), with ACPs positioned on specific auricular sites according to the standard protocol to ensure reproducibility.

Pain assessment

Pain intensity was evaluated using a standardized pinch test. The test site was located approximately 10 cm proximal to the lateral femoral epicondyle. Although lipedema pain can be present in several anatomical regions (e.g., arms, buttocks, abdomen), the lateral thigh is among the most consistently and intensely affected sites [5]. Choosing this location provided not only a reproducible landmark but also a clinically representative measure of lipedema pain, minimizing heterogeneity that would have arisen from comparing regions with variable tissue composition and symptom expression. Focusing on a single standardized site minimized variability related to differences in fat distribution, edema, or local tissue characteristics in other areas, thus improving consistency and reliability of measurements. Pain intensity during the pinch was recorded on a 10-point visual analog scale (VAS), ranging from 0 (no pain) to 10 (worst imaginable pain).

Data analysis

All statistical analyses were performed using IBM SPSS Statistics for Windows, Version 22 (Released 2013; IBM Corp., Armonk, New York, United States). Data normality was assessed with the Shapiro-Wilk test. Paired t-tests were used to compare VAS scores before and after both sham and real NPO sessions. As a sensitivity analysis, pre-post comparisons were also tested with the Wilcoxon signed-rank test. Effect sizes were calculated using Cohen’s d, and 95% confidence intervals (CI) were reported. Descriptive statistics are presented as means ± standard deviations (SD). A two-tailed p-value < 0.05 was considered statistically significant.

## Results

Baseline characteristics

The mean age of participants was 49.3 ± 11.2 years. Distribution by stage included stage 2 (n = 28), stage 3 (n = 37), stage 4 (n = 14), and stage 5 (n = 4). All reported chronic pain with baseline mean VAS > 7 in all subgroups. Baseline algometric pain was uniformly high (mean ± SD: 7.41 ± 0.53). A small number of pre-sham observations reached 10/10, without materially affecting dispersion. After sham NPO, no statistically or clinically significant changes were observed (p > 0.05). In contrast, real NPO produced a mean pain reduction exceeding 3.5 points (mean ± SD: -3.58 ± 0.62; p < 0.001) across all disease stages (n = 83).

**Table 1:** Baseline characteristics by lipedema stage

Stage	n	Age (years) mean ± SD	Baseline VAS mean ± SD
2	28	48.2 ± 10.8	7.40 ± 0.52
3	37	49.6 ± 11.5	7.42 ± 0.55
4	14	50.1 ± 12.0	7.41 ± 0.51
5	4	50.8 ± 10.9	7.43 ± 0.56
Total	83	49.3 ± 11.2	7.41 ± 0.53

Effect of sham NPO

**Figure 1:**
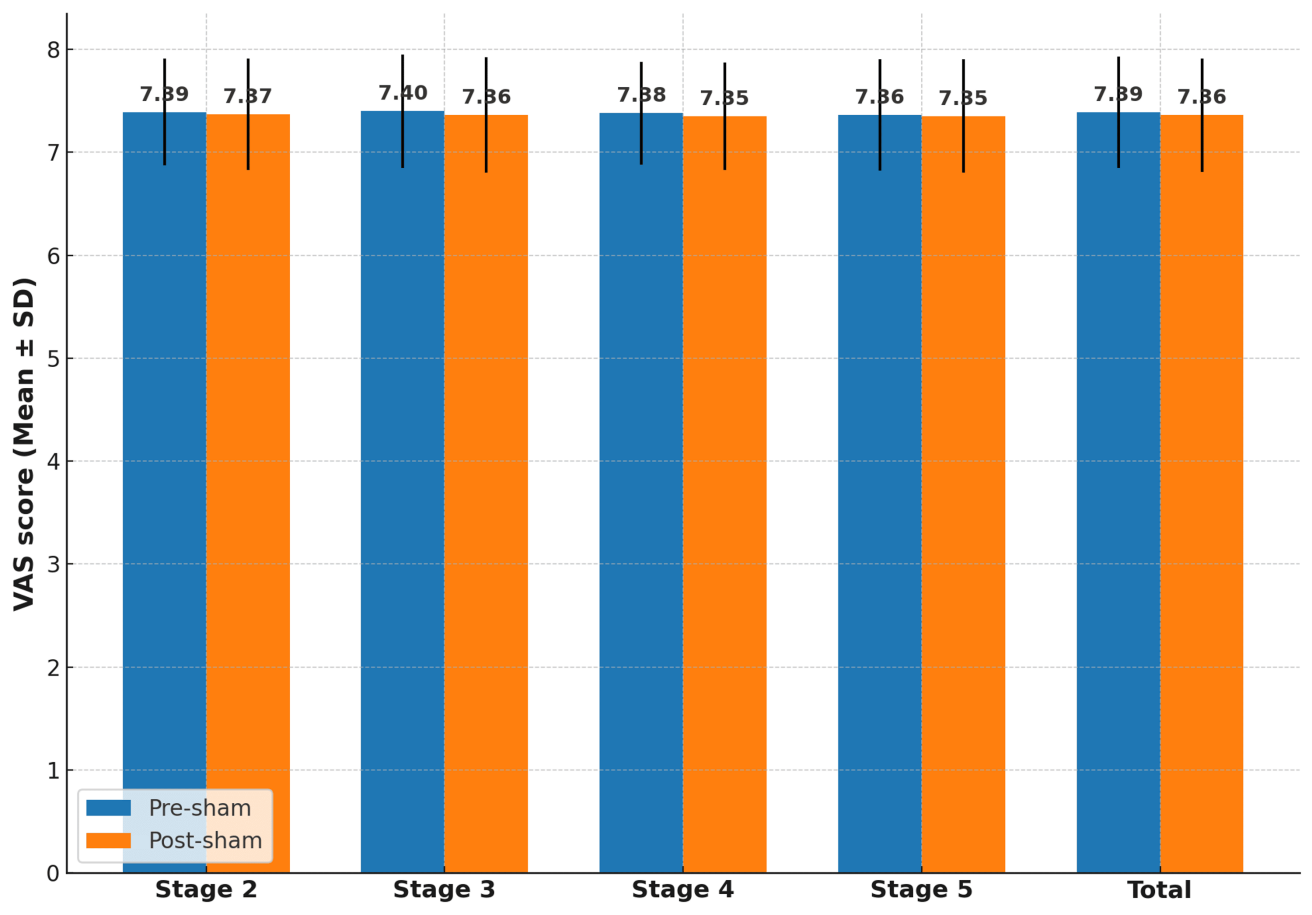
Mean ± SD VAS scores before and after sham NPO across all stages SD: standard deviation; VAS: visual analog scale; NPO: Neuro Postural Optimization The bar chart depicts mean values with error bars representing standard deviations. Pre- and post-sham measurements for each stage (2–5) are shown side by side, with the total group mean displayed separately. Minimal changes are visible across all stages, and statistical analysis confirms the absence of significant differences (p > 0.05), indicating no relevant placebo effect

VAS scores remained essentially unchanged after sham NPO (7.39 ± 0.54 pre-sham vs 7.36 ± 0.55 post-sham; p = 0.47, d = 0.05). Figure 1 shows the minimal variation, confirming no clinically relevant placebo effect.

Effect of real NPO

**Figure 2:**
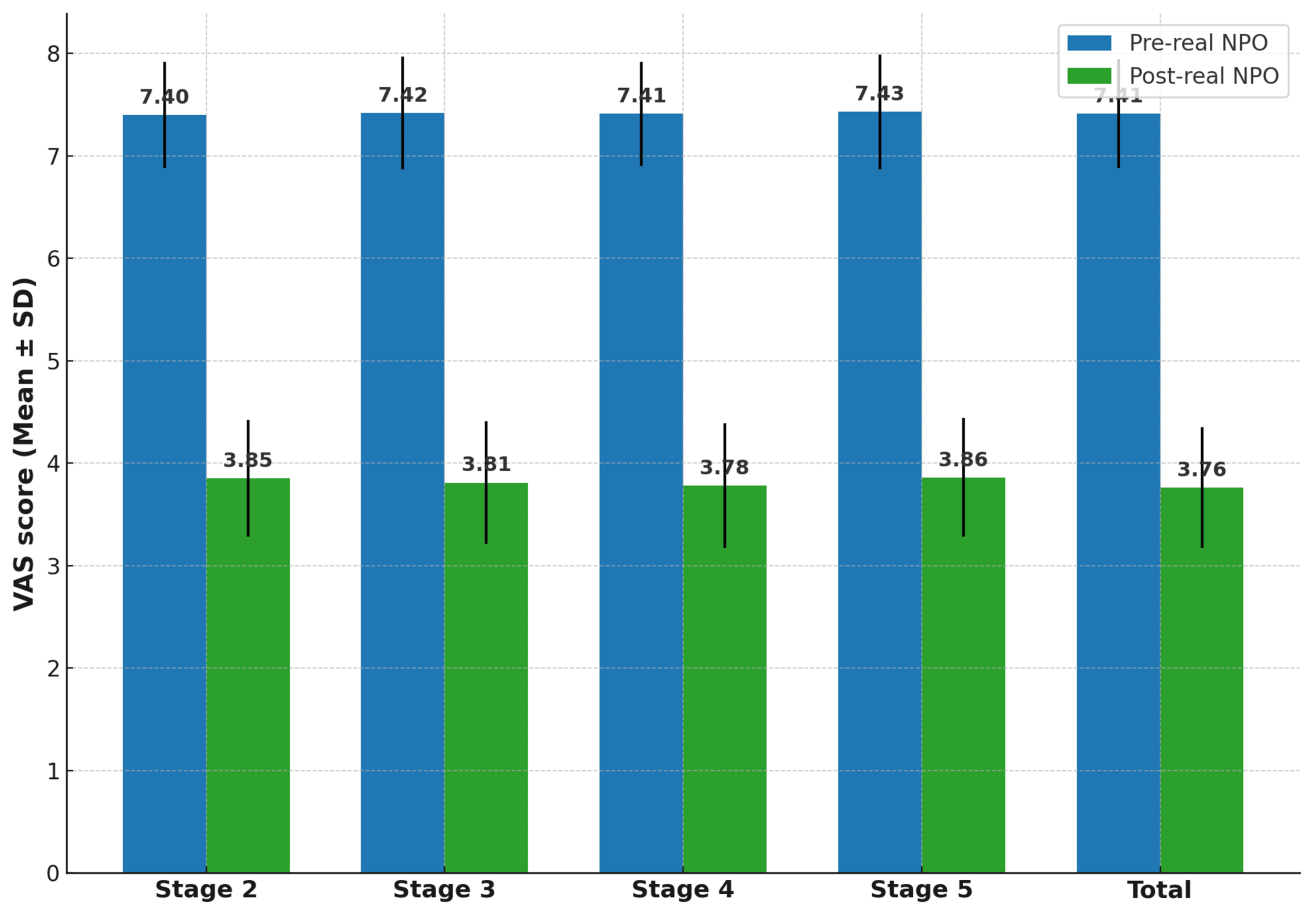
Mean ± SD VAS scores before and after real NPO across all stages, showing substantial and consistent reduction in pain perception SD: standard deviation; VAS: visual analog scale; NPO: Neuro Postural Optimization

Following real NPO, VAS decreased by 3.65 points on average (95% CI: -3.79 to -3.51), p < 0.001; Cohen’s dz = 5.88 (95% CI: 4.85-6.92). This reduction was consistent across all stages, with absolute mean decreases of 3.55 (stage 2), 3.61 (stage 3), 3.63 (stage 4), and 3.57 (stage 5). Table 2 summarizes pre- and post-treatment scores by stage, and Figure 2 illustrates the substantial reduction across the cohort.

**Table 2:** VAS scores pre- and post-sham, pre- and post-real NPO by stage SD: standard deviation; VAS: visual analog scale; NPO: Neuro Postural Optimization

Stage	Pre-sham VAS mean ± SD	Post-sham VAS mean ± SD	Δ Sham	Pre-real NPO VAS mean ± SD	Post-real NPO VAS mean ± SD	Δ Real NPO
2	7.39 ± 0.52	7.37 ± 0.54	-0.02	7.40 ± 0.52	3.85 ± 0.57	-3.55
3	7.40 ± 0.55	7.36 ± 0.56	-0.04	7.42 ± 0.55	3.81 ± 0.60	-3.61
4	7.38 ± 0.50	7.35 ± 0.52	-0.03	7.41 ± 0.51	3.78 ± 0.61	-3.63
5	7.36 ± 0.54	7.35 ± 0.55	-0.01	7.43 ± 0.56	3.86 ± 0.58	-3.57
Total	7.39 ± 0.54	7.36 ± 0.55	-0.03	7.41 ± 0.53	3.76 ± 0.59	-3.65

**Figure 3:**
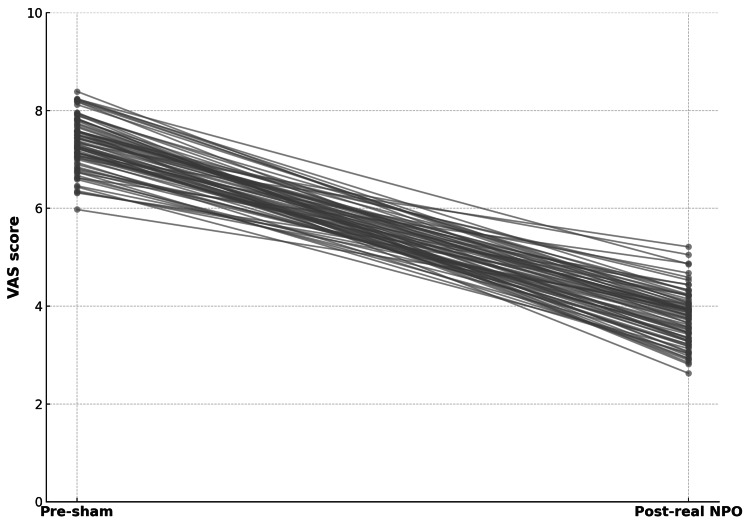
Individual patient trajectories in VAS scores from pre-sham to post-real NPO, illustrating uniform and marked pain reduction in all participants VAS: visual analog scale; NPO: Neuro Postural Optimization

All participants achieved at least a 30% reduction in VAS score following the real NPO treatment. A total of 78% experienced a reduction of 50% or greater, while 42% achieved a reduction of 60% or greater. Responder rates were similar across all lipedema stages, indicating that the magnitude of pain relief was independent of disease severity. The analysis of individual patient data (Figure 3) shows a consistent downward shift in VAS scores from pre-sham to post-real NPO for every participant, confirming the uniformity of the analgesic effect at the individual level.

Adverse events

No adverse events or discomfort were reported during either the sham or the active NPO sessions.

## Discussion

This sham-controlled pre-treatment study provides compelling evidence that the REAC NPO protocol [1,4] can produce an immediate and clinically significant reduction in pain perception in women with advanced lipedema (stages 2-5) [9], most of whom had previously failed to respond to conventional therapies [21]. By incorporating a sham phase in which all procedural aspects were identical but no active REAC signal was delivered, we were able to exclude expectancy or procedural bias as explanations for the observed analgesic effect. The lack of change after sham NPO contrasted sharply with the uniform and statistically robust reduction in VAS scores after real NPO, with mean decreases exceeding 3.5 points on a 10-point scale across all disease stages. This magnitude of change is well above the threshold for clinical relevance in chronic pain conditions [22].

The use of the pinch test [23] is particularly noteworthy, as it is a clinically validated and condition-specific tool for provoking and quantifying pain in lipedema. Its inclusion reinforces the reliability of the observed effects. FD is a congenital neuropsychophysical condition characterized by maladaptive postural responses to environmental stimuli [1]. In lipedema, these maladaptive responses may exacerbate venous-lymphatic overload, tissue inflammation, and microvascular congestion, intensifying pain and disease progression [24]. By correcting FD through targeted bioelectrical modulation, the NPO protocol may restore physiological patterns of postural control and sensory integration, thereby reducing biomechanical stress, improving neuromuscular coordination, and mitigating neuroinflammatory signaling [4]. The consistency of the effect across stages supports the hypothesis that central mechanisms, rather than the extent of peripheral adipose tissue changes, are key contributors to pain in lipedema [25].

Beyond the immediate analgesic response, the sudden pain reduction may also have psychological implications [26]. Pain is a major factor limiting mobility, emotional well-being, and quality of life in lipedema [27]. The ability of NPO to induce rapid relief may thus positively impact confidence, motivation, and psychosocial functioning. Importantly, NPO produces a binary outcome with respect to FD correction: either the maladaptive asymmetry resolves immediately or it does not [1]. While this inherently allows the examiner to distinguish real from sham procedures, the subjective pain reporting by patients, which was the primary outcome, remains unbiased. Moreover, the fixed sham→real sequence was necessary, as once FD is corrected, it cannot be re-induced to test sham validity [1].

The safety profile was excellent, with no adverse events reported, consistent with previous REAC studies [28]. Strengths of this study include its sham-controlled design, the standardized assessment of pain, and the homogeneous distribution of baseline pain scores across disease stages.

However, several limitations must be acknowledged. The retrospective single-center design limits generalizability and precludes control over all potential confounding variables. The absence of systematic recording of comorbidities, although no major conditions were identified, may have overlooked subtle contributors to pain variability. The fixed sham→real sequence, while unavoidable for the reasons explained, prevents assessment of prolonged placebo effects. In addition, the study did not include long-term follow-up, functional outcome measures, or quality-of-life assessments, which would be necessary to evaluate the persistence and broader clinical impact of the analgesic effect. These limitations are acknowledged to provide a balanced interpretation of the study’s strengths and weaknesses.

The sample size (n = 83) is relatively large for a single-center observational study and sufficient to demonstrate statistically robust effects, though prospective multicenter investigations with longer follow-up and broader outcome measures are needed to confirm generalizability and to better characterize the clinical relevance of FD correction in lipedema. Nevertheless, the consistency of patient-reported improvements in gait fluidity, stability, and reduced stiffness suggests a broader impact beyond pain relief, warranting further investigation.

Clinical and mechanistic implications

NPO is an extensively documented intervention [1,4]. The present findings indicate that lipedema should not be viewed solely as a disorder of adipose tissue accumulation but also as a condition aggravated by maladaptive neuropsychomotor patterns rooted in FD [10]. By correcting this upstream dysfunction, NPO directly addresses a hidden but crucial contributor to venous-lymphatic overload, tissue stress, and pain amplification. While the primary objective of this study was clinical and symptomatic, mechanistic aspects of REAC technology, including bioelectrical reprogramming, positive epigenetic modulation, and neuronal circuit reorganization, have been extensively demonstrated in previous work [4,28]. Future research should further integrate neurophysiological assessments, such as quantitative sensory testing [25], to clarify the role of FD correction in the multifactorial pain experience of lipedema.

## Conclusions

FD may play a contributory role in the development and progression of lipedema by promoting maladaptive postural and behavioral responses that aggravate tissue stress and pain. Correction of FD via REAC NPO produced an immediate and significant reduction in pain intensity in this cohort of lipedema patients. These findings highlight the potential role of NPO as a noninvasive, neurobiological modulatory intervention that addresses an upstream pathophysiological mechanism in lipedema. However, due to the retrospective single-center design, absence of long-term follow-up, and lack of broader outcome measures, these conclusions should be interpreted as applying only to the short-term effects observed. Further multicenter, prospective, and longitudinal studies are warranted to confirm these results, determine their long-term clinical significance, and explore the broader functional and quality-of-life implications of NPO in lipedema management.
